# World Health Organization Recommendation for Using Uterine Balloon Tamponade to Treat Postpartum Hemorrhage

**DOI:** 10.1097/AOG.0000000000004674

**Published:** 2022-02-02

**Authors:** Andrew D. Weeks, Oluwarotimi Ireti Akinola, Melania Amorim, Brendan Carvalho, Catherine Deneux-Tharaux, Tippawan Liabsuetrakul, Martin Meremikwu, Suellen Miller, Ashraf Nabhan, Mari Nagai, Hayfaa Wahabi, Dilys Walker

**Affiliations:** University of Liverpool, Liverpool, United Kingdom; the Society of Gynaecology and Obstetrics in Nigeria (SOGON), Abuja, Nigeria; Instituto Paraibano de Pesquisa Professor Joaquim Amorim Neto and Instituto de Medicina Integral Professor Fernando Figueira, Paraiba, Brazil; the Division of Obstetric Anesthesia, Department of Anesthesiology, Perioperative and Pain Medicine, Stanford University School of Medicine, Stanford, California; the Obstetrical, Perinatal and Pediatric Epidemiology Research Team, Centre of Research in Epidemiology and Statistics, Sorbonne, Paris, France; the Faculty of Medicine, Prince of Songkla University, Hat Yai, Songkhla, Thailand; the University of Calabar, Calabar, Nigeria; the Safe Motherhood Program, Department of Obstetrics, Gynecology and Reproductive Sciences, and the Department of Obstetrics, Gynecology & Reproductive Sciences, University of California, San Francisco, San Francisco, California; the Department of Obstetrics and Gynecology, Ain Shams University, Cairo, Egypt; the Bureau of International Health Cooperation, National Centre for Global Health and Medicine, Tokyo, Japan; and Evidence-based Healthcare and Knowledge Translation, College of Medicine, King Saud University, Riyadh, Saudi Arabia.

## Abstract

The World Health Organization (WHO) recently published a new recommendation on the use of the uterine balloon tamponade for the treatment of postpartum hemorrhage. The recommendation that uterine balloon tamponade should be used only where there is already access to other postpartum hemorrhage treatments (including immediate recourse to surgery) has proved controversial. It is especially problematic for those working in low-level health care facilities in under-resourced settings, where there are already programs that have introduced low-cost uterine balloon tamponade devices for use, even in settings where recourse to surgical interventions is not possible. However, there are now two separate randomized trials that both unexpectedly show unfavorable outcomes in these settings when a condom catheter uterine balloon tamponade device was introduced. Considering the balance of potential benefits and these safety concerns, the WHO postpartum hemorrhage guideline panel therefore recommends that uterine balloon tamponade should be used only in contexts where other supportive postpartum hemorrhage interventions are available if needed.

## Abstract 2

The World Health Organization recommends that balloon tamponade should be used only where other supportive interventions are available if needed.

In May 2021, as part of their living guidelines process, the World Health Organization (WHO) published its updated recommendations on the use of uterine balloon tamponade for the treatment of postpartum hemorrhage.^1^ The past decade has seen several important new studies on uterine balloon tamponade,^2,3^ and the update was, therefore, eagerly awaited. However, the WHO's new recommendation that the use of uterine balloon tamponade is recommended only in situations where standard postpartum hemorrhage care, including surgical intervention and blood for transfusion, is accessible has caused concern. There are several large international initiatives successfully promoting uterine balloon tamponade use in under-resourced or lower-level health facilities,^4–6^ and there is concern that this updated recommendation could stall the progress they have made, confuse clinicians, and block the introduction of the program into new countries. This article explains the underlying rationale for the recommendation and provides guidance for clinicians, researchers, and program and policy makers in reproductive health care settings.

## THE 2021 WORLD HEALTH ORGANIZATION UTERINE BALLOON TAMPONADE RECOMMENDATION

At the outset, it is important to clarify exactly what the new guidelines state. The WHO Guideline Development Group has developed a context-specific recommendation, stating that:

Uterine balloon tamponade is recommended for the treatment of postpartum hemorrhage due to uterine atony after vaginal birth in women who do not respond to standard first-line treatment, provided the following conditions are met:

- Immediate recourse to surgical intervention and access to blood products is possible if needed.
- A primary postpartum haemorrhage first-line treatment protocol (including the use of uterotonics, tranexamic acid, intravenous fluids) is available and routinely implemented.
- Other causes of postpartum haemorrhage (retained placental tissue, trauma) can be reasonably excluded.
- The procedure is performed by health personnel who are trained and skilled in the management of postpartum haemorrhage, including the use of uterine balloon tamponade.
- Maternal condition can be regularly and adequately monitored for prompt identification of any signs of deterioration.^1^

## EVIDENCE OF EFFICACY AND SAFETY FROM THE LITERATURE REVIEWS

As is common in reviews of any scientific evidence, concerns regarding the potential for bias in nonrandomized studies and case series led the Guideline Development Group to focus primarily on evidence from randomized trials, considering not only efficacy but concerns over safety. Although there are very few high-quality data, the evidence synthesis considered by the Guideline Development Group is in line with a Cochrane systematic review of mechanical and surgical postpartum hemorrhage management. This review, based on very low certainty evidence, concludes that, “the finding that intrauterine tamponade may increase total blood loss >1,000 mL suggests that introducing condom‐balloon tamponade into low‐resource settings on its own without multi‐system quality improvement does not reduce postpartum hemorrhage deaths or morbidity.”^7^ The reported potential harm shown in the randomized trials is an important finding that cannot be ignored. One of the key decision-making principles of the Guideline Development Group is the balance between the desirable and undesirable effects of the intervention. It is critical, therefore, that WHO does not recommend an intervention with potential safety concerns for global use.

In addition to the Cochrane review, there is a second comprehensive systematic review by Suarez et al^8^ that suggests a very high success rate for the uterine balloon tamponade. However, the evidence of high uterine balloon tamponade success was based largely on nonrandomized studies and case series, which made up 92% of the 91 included studies. The fallacy of an apparently high success rate in the absence of a control group is a well-recognized problem with postpartum hemorrhage owing to the spontaneous resolution of many postpartum hemorrhages. For example, we saw dramatic initial benefits with misoprostol for severe unresponsive postpartum hemorrhage in uncontrolled trials,^9^ only for later randomized trials to reveal that it was less effective than oxytocin for prophylaxis and of no additional benefit for treatment when oxytocin had already been administered.^10–12^

Another systematic review, examined during the Guideline Development Group meetings but published only later, shows a third way of examining the evidence.^13^ That review included all controlled studies (whether randomized or not) of uterine balloon tamponade compared with standard care for postpartum hemorrhage after vaginal birth and had a composite outcome of death or need for surgical intervention. Only four studies provided analyzable data, and the quality of evidence was reported as “low to very low certainty.” There was some evidence of increased need for further intervention after uterine balloon tamponade, but the authors conclude that the overall effect of uterine balloon tamponade was unclear.

The Guideline Development Group considered the evidence from all three systematic reviews, which is reflected in the uterine balloon tamponade recommendation that was formulated. The Guideline Development Group also took into consideration that the randomized trial evidence all came from studies using improvised condom catheter uterine balloon tamponade devices, whereas there are now several low-cost commercial balloon devices available, with limited evidence regarding effectiveness and cost.^14^

## CONCERNS REGARDING THE SPECIFIC CONTEXT FOR THE USE OF UTERINE BALLOON TAMPONADE

Two specific concerns have been voiced since the guideline was developed. These regard the statements that uterine balloon tamponade is recommended both, where “Immediate recourse to surgical intervention and access to blood products is possible if needed[,]” and where “…first-line treatment protocol (including the use of uterotonics, tranexamic acid, intravenous fluids) is available and routinely implemented.”

The concern centers around the plight of women in the community and at health care facilities that provide only basic emergency obstetric and newborn care, where blood transfusions and surgery are not available. However, it is at these very health care–provision levels that the available randomized controlled trials of condom catheter uterine balloon tamponade by Anger et al^2^ and Dumont et al^3^ were conducted. Although imperfect, these are the only randomized trials available comparing uterine balloon tamponade with standard care, and both of these trials found not only no benefit, but a worsening of outcomes with the introduction of uterine balloon tamponade devices. In the Dumont study, the rate of total blood loss greater than 1,000 mL was significantly increased in patients who had tamponade (relative risk 1.52, 95% CI 1.15–2.00).^3^ In the Anger study, the incident rate of postpartum hemorrhage–related surgery or maternal death was also significantly increased in those clusters trained in use of the condom catheter (incident rate ratio 4.08; 95% CI 1.07–15.58).^2^ Although the reason for these findings is not entirely clear, the Guideline Development Group took the results seriously—it would be negligent to ignore the findings in a controlled setting just because they gave an unexpected result. Indeed, the Guideline Development Group is supported in this view by the authors of the Anger et al randomized trial. In the discussion, the authors state:

“These findings suggest that interventions such as UBT [uterine balloon tamponade] may have limited effectiveness in improving maternal outcomes when introduced into resource‐constrained health systems with unreliable access to other essential components of emergency care. Because the management of refractory haemorrhage requires a response that is complex and is thus dependent on other health system capacities, it is difficult to judge the effect of UBT introduction and to generalise our study findings. More encouraging results of UBT implementation may be observed elsewhere with more favourable environments (e.g. reliable blood supply), with a different UBT device or with a longer observation period.”^2^

## OPTIONS FOR WORLD HEALTH ORGANIZATION RECOMMENDATIONS

In the WHO guideline decision-making process, the Guideline Development Group has four options: to recommend in favor of the intervention, to recommend against the intervention, to recommend only in specific contexts, or to recommend only in the context of rigorous research. Given the randomized trial evidence showing not only a lack of benefit but evidence of harm, the Guideline Development Group felt unable to provide an unconditional recommendation. It therefore formulated a context-specific (otherwise described as “conditional”) recommendation for use of uterine balloon tamponade, considering the potential safety issues identified in the settings where randomized trials have shown evidence of harm.

The preconditions agreed on by the Guideline Development Group for uterine balloon tamponade use seem to have been misinterpreted as denying women access to an effective intervention at lower-level health care facilities and in the community. On the contrary, it does not recommend against the use of uterine balloon tamponade in those resource-limited settings but highlights specific conditions to optimize the quality of postpartum hemorrhage care and guarantee safety at all levels of the health care system. This global recommendation is expected to serve as a benchmark for local adoption and adaptation at the country, regional, and subregional levels. As stated in the implementation considerations of the uterine balloon tamponade recommendation, program managers and policy makers should adapt the recommendations into their own context:

“The recommendation should be adapted into documents and tools that are appropriate for different locations and contexts, to meet the specific needs of each country and health service. Modifications to the recommendation, where necessary, should be justified in an explicit and transparent manner.”^1^

This provides an opportunity for continued use of uterine balloon tamponade in those contexts where all preconditions may not be met but health care policy makers and clinicians believe it could safely meet the needs of their health care system. Maternal health care clinicians who are currently using uterine balloon tamponade in facilities without access to surgical interventions and blood products will need to review their practice in light of these recommendations. Any modifications should be justified in a way such that the target end users of uterine balloon tamponade are fully aware of any compromises being made to build appropriate safety nets within the health care system.

## OPTIONS FOR HEALTH MANAGERS AND CLINICIANS WORKING AT CENTERS CURRENTLY IMPLEMENTING UTERINE BALLOON TAMPONADE

### Use of Uterine Balloon Tamponade as Part of an Upscaling of Postpartum Hemorrhage Care

There is widespread agreement that it is appropriate to implement and reinforce first-line measures before more invasive techniques, and settings without access to these interventions should focus first on implementing these before uterine balloon tamponade.^15^ This principle is reflected in the WHO recommendation of uterine balloon tamponade for treatment of postpartum hemorrhage in women who do not respond to standard first-line treatment (ie, refractory postpartum hemorrhage). It is therefore imperative that these first-line measures (including uterotonics, tranexamic acid, and intravenous fluids) are routinely implemented first before inserting a uterine balloon tamponade device. Management of refractory postpartum hemorrhage includes a range of interventions, such as compressive measures (aortic compression or bimanual uterine compression), the nonpneumatic anti-shock garment, blood transfusion, and surgical interventions.^15^ All are critical lifesaving interventions for the prevention of postpartum hemorrhage–related maternal deaths. Organizers of maternity care should consider how all of these interventions can best be made available for births outside of a hospital, either at the low-level health care facility or after emergency transport. The recommendations suggest that uterine balloon tamponade devices should not be introduced as a standalone intervention without access to other lifesaving measures, but as part of a wider range of quality improvement measures that includes access to surgical interventions and blood transfusion if needed.

### Use of Uterine Balloon Tamponade in the Context of Research

In health care facilities where the preconditions for use of uterine balloon tamponade cannot be met, clinicians could still introduce it in the context of research. This is called for in the systematic review as well as in the remarks accompanying the WHO recommendation. Ideally this would be done as a high-quality randomized trial in low-level facilities where first-line treatment measures are reinforced in all facilities and randomized facilities receive a commercially developed uterine balloon tamponade device, but it could also include individual hospitals exploring the local effects of uterine balloon tamponade introduction. Any individual hospitals conducting research should ensure that they collect data on postpartum hemorrhage outcomes both with and without uterine balloon tamponade use, so that comparative data on comparable women are available, and they should seek to close the system gaps that may have led to the uterine balloon tamponade failures.

## MOVING FORWARD

The divergence between the personal experiences of clinicians (as reflected in observational studies and case reports) and the controlled research trials is not easy to deal with on a personal, professional organization, or system level. It remains unclear why the randomized trials of uterine balloon tamponade have failed to show benefit, or indeed why they appear to show harm. The uncertainty as to whether it was due to the device, training, or setting can be disentangled only by further high-quality randomized trials conducted using commercially available uterine balloon tamponade devices. Such a trial is currently being organized under the leadership of the WHO in Vietnam. This study will examine a variety of second-line techniques for treating postpartum hemorrhage, including a commercial balloon device, uterine suction, and the local standard of Foley balloon catheter. This will help determine whether the poor results in the randomized trials to date are simply a result of the use of the clinician-assembled condom catheter, the capacity level in low-resource settings, or other factors. In the meantime, there must be a focus on the delivery of high-quality first-line postpartum hemorrhage care in lower-level facilities, improving the transport of those who need higher-level care, and the rapid delivery of high-quality secondary care for those transferred. Without these basics in place, it will be difficult to save lives even if uterine balloon tamponade is finally proven to be effective in low-level facilities. It is worth stating again that, in postpartum hemorrhage, there are sadly no magic bullets and few shortcuts.
